# Genomic landscape of immune checkpoint inhibitor-induced aplastic anemia: a case report

**DOI:** 10.3389/fonc.2026.1801452

**Published:** 2026-05-20

**Authors:** Nobuyuki Takahashi, Kenichi Harano, Chikako Funasaka, Hiromichi Nakajima, Chihiro Kondoh, Nobuaki Matsubara, Ako Hosono, Yoichi Naito, Michiko Nagamine, Hiroshi Tanabe, Takao Fujisawa, Yoshiaki Nakamura, Hideaki Bando, Takayuki Yoshino, Junichiro Yuda, Toru Mukohara

**Affiliations:** 1 Department of Medical Oncology, National Cancer Center Hospital East, Kashiwa, Japan; 2 Minamiakatsuka Clinic, Mito, Japan; 3 Department of Experimental Therapeutics, National Cancer Center Hospital East, Kashiwa, Japan; 4 Department of General Internal Medicine, National Cancer Center Hospital East, Kashiwa, Japan; 5 Department of Pathology and Clinical Laboratories, National Cancer Center Hospital East, Kashiwa, Japan; 6 Department of Gynecology, National Cancer Center Hospital East, Kashiwa, Japan; 7 Translational Research Support Office, National Cancer Center Hospital East, Kashiwa, Japan; 8 Department of Head and Neck Medical Oncology, National Cancer Center Hospital East, Kashiwa, Japan; 9 Department of Gastrointestinal Oncology, National Cancer Center Hospital East, Kashiwa, Japan; 10 Department of Hematology and Oncology, National Cancer Center Hospital East, Kashiwa, Japan

**Keywords:** aplastic anemia, case report, clonal hematopoiesis, immune checkpoint inhibitor, immune-related adverse events

## Abstract

Hematological immune-related adverse events caused by immunotherapy are relatively uncommon. Aplastic anemia triggered by immune checkpoint inhibition is rare and its genomic characteristic remains largely unexplored. This case report describes a 42-year-old female with metastatic cervical cancer who experienced aplastic anemia caused by pembrolizumab. Through a nationwide genomic screening study, MONSTAR-SCREEN-2, we examined genomic landscape of biopsied tissue and circulating tumor DNA. The patient harbored human leukocyte antigen-A*0201 haplotype, which is known as a risk allele of idiopathic acquired aplastic anemia. Clonal hematopoiesis (CH) of myeloid cancer candidate gene *DNMT3A* c.1429 + 1G>A was newly detected in the buffy-coated white blood cell sample at the time of aplastic anemia diagnosis. This case report contributes to further progress of genomic research of immune-related aplastic anemia.

## 1 Introduction

Immune checkpoint inhibitors (ICIs) have revolutionized cancer care by enhancing antitumor immunogenicity through the blockade of cytotoxic T-lymphocyte-associated protein 4, programmed cell death protein 1, or programmed cell death ligand 1 signaling. However, ICIs can trigger unique adverse events via autoimmune mechanisms, termed immune-related adverse events (irAE) (1). These irAEs can affect various organs, including the hematopoietic system. Hematological irAEs by ICIs are relatively uncommon compared to those affecting other organs, representing a small fraction of all irAEs (2). ICI-induced aplastic anemia rarely occurs, and the genomic characteristics of this condition remain largely unexplored.

## 2 Case description

**Figure 1:**
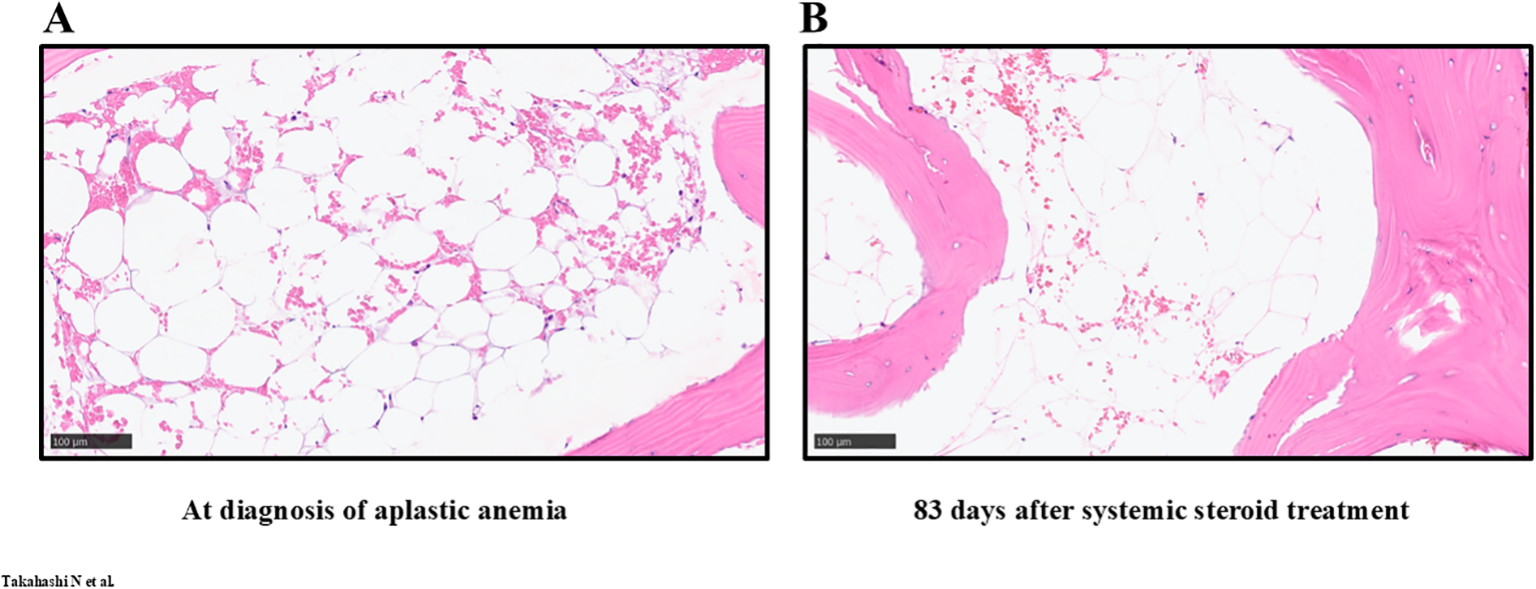
Sequential pathological images of bone marrow. **(A)** Hematoxylin and eosin-stained pathological images at the time of aplastic anemia, 200x. **(B)** Hematoxylin and eosin-stained pathological images obtained 83 days after the initiation of systemic steroid treatment, 200x.

**Figure 2:**
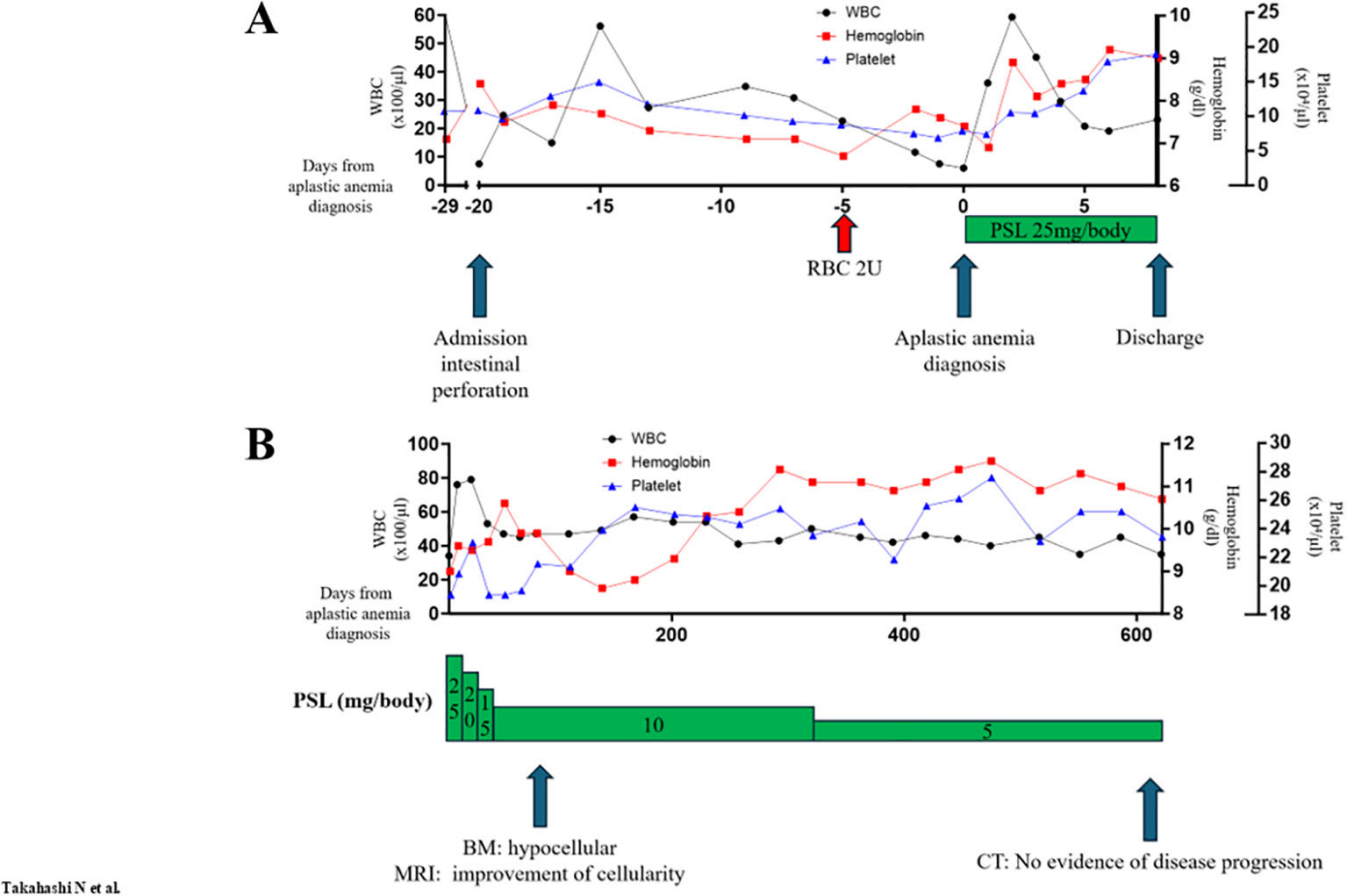
Clinical course of immunotherapy-induced aplastic anemia. **(A)** Peripheral blood cell counts during admission. **(B)** Peripheral blood cell counts as outpatient. Days on the x-axis show the days from the diagnosis of immunotherapy-induced aplastic anemia. WBC, white blood cell; RBC, red blood cell transfusion; PSL, prednisolone; BM, bone marrow biopsy; MRI, magnetic resonance imaging; CT, computed tomography.

A 42-year-old female, previously treated with definitive chemoradiotherapy for clinical stage IIIC cervical squamous cell carcinoma, experienced recurrence in her cervical, mediastinal, axillary, and para-aortic lymph nodes four months post-treatment. The patient received palliative systemic therapy comprising carboplatin (area under the free carboplatin plasma concentration vs. time curve 5, day 1), paclitaxel (175 mg/m^2^, day 1), bevacizumab (15 mg/kg, day 1), and pembrolizumab (200 mg/body, day 1) in a 21-day cycle, resulting in partial responses. On day 10 of cycle 5, she was hospitalized due to intestinal perforation and managed conservatively with fasting and intravenous antibiotics following consultation with surgeons and gynecologists, concerning potential complications by recent bevacizumab administration. Her symptoms resolved within days. Thirteen days after admission, she experienced spontaneous epistaxis. Laboratory tests revealed pancytopenia without abnormal cells or hemolysis on peripheral blood smear (**Table 1**). Virological examination and autoantibody screening did not identify the etiology of her pancytopenia (**Supplementary Table 1**). Magnetic resonance imaging (MRI) of her vertebrae exhibited high- and low-intensity signals on T1-weighted and T2-weighted images, respectively. Quantitative Dixon fat suppression and short inversion time inversion recovery long echo time images displayed high and low intensities, respectively (**Supplementary Figure 1A**). A bone marrow biopsy from her iliac crest revealed severe hypocellular marrow without metastatic carcinoma (**Figure 1A**). Suspecting severe immune-mediated aplastic anemia induced by pembrolizumab, prednisolone at 0.5 mg/kg/day (equivalent to 25 mg/body/day) was initiated. A relatively low dose of systemic steroid (recommended dose: 1–2 mg/kg) (3) was chosen for severe aplastic anemia related to ICIs, considering her peritonitis and intestinal perforation. A single dose of granulocyte-colony stimulating factor (filgrastim 75 μg/body) was also administered. Her peripheral blood counts were immediately improved. The patient was discharged eight days after starting systemic steroid therapy (**Figure 2A**). An MRI of her vertebrae 83 days after initiation of systemic steroid therapy showed increased signal intensity on short inversion time inversion recovery and long echo time sequences compared with findings at the time of aplastic anemia diagnosis, suggesting an improvement in bone marrow cellularity (**Supplementary Figure 1B**). However, despite these imaging changes, the bone marrow continued to demonstrate severe hypoplasia (**Figure 1B**). The prednisolone dose was tapered to 5 mg/body/day over 600 days after systemic steroid initiation, and her peripheral blood count was sustained with the levels of complete response (defined by normal hemoglobin, absolute neutrophil count of more than 1.5 x 10^9^/l, and platelet count of more than 150 x 10^9^/l) (4). Serial computed tomography continuously showed ongoing responses even if systemic chemoimmunotherapy had not been resumed (**Figure 2B**).

**Table 1:** Laboratory characteristics at the diagnosis of immunotherapy-induced aplastic anemia.

Hematology			Chemistry			Coagulopathy		
WBC	600	/μl	TP	6.6	g/dl	PT	13.9	sec
Baso	0	%	Alb	3.0	g/dl	PT-INR	1.2	
Eosino	6	%	T-Bil	0.2	mg/dl	APTT	32.4	sec
Promyelo	0	%	AST	18	U/L	Fibrinogen	559	mg/dl
Myelo	0	%	ALT	14	U/L	FDP	20.8	μg/ml
Meta	0	%	ALP	106	U/L	D-dimer	5.7	μg/ml
Stab	1	%	LDH	154	IU/L	Factor VIII activity	57	%
Seg	77	%	BUN	16.0	mg/dL			
Lymph	10	%	Cre	0.57	mg/dl	Endocrinology		
Atyp.Lym	0	%	Cl	93	mEq/L	TSH	0.1	μIU/ml
Mono	6	%	Na	137	mEq/L	Free-T3	4.23	pg/ml
Other	0	%	K	3.7	mEq/L	Free-T4	2.16	ng/dl
Blast	0	%	Cl	98	mEq/L	ACTH	105	pg/ml
Eryblast	0	%	CRP	0.15	mg/dl	Cortisol	11.3	μg/dl
RBC	228	10^4^/μl	Fe	46	μg/dl			
Hb	7.6	g/dl	UIBC	198	μg/dl			
HCT	23.2	%	TIBC	244	μg/dl			
MCV	99.1	fl	Transferrin saturation	19				
MCH	32.5	Pg	Haptoglobin	516	mg/dl			
MCHC	32.8	%	Erythropoietin	28.4	mIU/ml			
Reticulocyte	1.25	%	Thrombopoietin	28.6	Fmol/ml			
Plt	7.0	10^4^/μl	Ferritin	449	ng/ml			
			Vit. B12	985	pg/ml			
			Folate	3.9	ng/ml			
			Ceruloplasmin	67	mg/dl			

Through a nationwide genomic screening study, MONSTAR-SCREEN-2 (5, 6), we examined the genomic profiles of the patient’s primary cervical lesion at diagnosis, the longitudinal circulating tumor DNA collected before starting systemic chemoimmunotherapy, and at the time of diagnosis of ICI-induced aplastic anemia. We utilized Caris MI CancerSeek for tumor tissues and Caris ASSURE for plasma samples (Caris Life Sciences, USA), both of which are next-generation sequencing assays (7, 8). MONSTAR-SCREEN-2 received approval from the Institutional Review Board of the National Cancer Center and was registered in the University Hospital Medical Information Network Clinical Trials Registry (protocol ID: UMIN000043899) following the ethical guidelines of the Declaration of Helsinki. Genomic profiling of tumor biopsies at the initial diagnosis revealed gene alterations commonly associated with cervical squamous cell carcinoma (9), including *ARID1A* p.V2152fs, *PTEN* p.R130*, *BAP1* p.R385*, and *KMT2D* p.P648fs. The patient also had the human leukocyte antigen (HLA)-A *02:01 haplotype. The genomic profile of her blood before commencing systemic chemo-immunotherapy mirrored that of her primary cervical lesion at diagnosis, indicating that liquid-based genomic profiling captured circulating tumor DNA. These alterations decreased when the patient was diagnosed with IO-induced aplastic anemia, suggestng partial responses to systemic treatment across the metastatic lesions. Clonal hematopoiesis (CH) of *DNMT3A* c.1429 + 1G>A was newly detected in the buffy-coated white blood cell sample at the time of aplastic anemia diagnosis (**Table 2**).

**Table 2:** Genomic profiling and HLA haplotype in the tumor and circulating cell tumor DNA.

Genomic alteration			
Tumor at diagnosis	VAF (%)		
*BAP1* p.R385*	28		
*PTEN* p.R130*	22		
*FCLN* p.H429fs	20		
*FBXW7* p.R465C	19		
*PTPN11* p.A72T	19		
*SMARCB1* p.R377C	19		
*NOTCH1* p.V1578del	18		
*KMT2D* p.P648fs	16		
*RAD50* p.N934fs	9		
*FGFR3* p.R248C	6		
ctDNA before treatment	VAF (%)	ctDNA at AA diagnosis	VAF (%)
*BAP1* p.R385*	8	*TCF7L2* c.1200+2T>C	1.2
*EP300* p.N1559fs	2.6	*TNFRSF14* p.Q182*	0.3
*PTEN* p.R130*	2.2		
*KMT2D* p.P648fs	1.9		
*ARID1A* p.V2152fs	1.3		
Clonal hematopoiesis			
ctDNA before treatment	Plasma/WBC VAF (%)	ctDNA at AA diagnosis	Plasma/WBC VAF (%)
None		*DNMT3A* c.1429+1G>A	0.3/0.6
HLA haplotype			
HLA-A	A*02:01, A*11:01		
HLA-B	B*15:01, B*51:01		
HLA-C	C*01:02, C*14:02		

Genomic profiling was performed using Caris MI Profile (7) for tumor tissues and Caris ASSURE (8) for plasma samples (Caris Life Sciences, USA). HLA, human leukocyte antigen; VAF, variant allele frequency; ctDNA, circulating tumor DNA; WBC, white blood cell.

## 3 Discussion

The ICIs can induce hematological irAE including aplastic anemia. Although the frequency of the hematological irAE is relatively fewer, several case reports or series of ICI-induced aplastic anemia have been reported (10, 11). However, to the best of our knowledge, this is the first case report evaluating genomic landscape of ICI-induced aplastic anemia through the nationwide genomic screening study MONSTAR-SCREEN-2 (5, 6).

The CH in *DNMT3A* was newly identified in the patient’s buffy-coated white blood cells when she was diagnosed with ICI-induced aplastic anemia. CH was not detected in the blood samples collected before initiating systemic treatment. CH of myeloid cancer candidate genes was detected in 47% of patients with idiopathic acquired aplastic anemia, including *DNMT3A*, *BCOR*, *PIGA*, and *ASXL1*. Although variant allele frequency of these CH is initially low (less than 10%), it subsequently expands after immunosuppressive treatment, suggesting bone marrow recovery and clonal expansion of myeloid cells with these CH (12). The development of *DNMT3A* CH suggests that the pre-existing hematopoietic stem cells with the mutation gain in a selective pressure survival under the immune-mediated cytotoxic attack induced by immune checkpoint inhibitors. Alternatively, the mutation works as a protective factor against the immune-mediated attack along with the obligation of normal hematopoietic stem cells. As the marrow recovers, myeloid cells with the mutation are developed in the recovery of the hematopoietic stem cell compartment (16). We could not evaluate genomic characteristics of circulating tumor DNA after systemic steroid treatment and recovery of peripheral blood counts because sample collection in such timepoint is not included in the MONSTAR-SCREEN-2 project, warranting further longitudinal genomic studies for such rare irAEs. The patient also harbored the HLA-A*02:01 allele. The HLA haplotype is a known risk allele for idiopathic-acquired aplastic anemia (13). These findings suggest that the genomic characteristics are similar to those of idiopathic acquired aplastic anemia.

Cytotoxic chemotherapies, such as carboplatin and paclitaxel, can induce myelosuppression. However, the bone marrow biopsy revealed severe hypocellularity with lacunar spaces filled with adipose tissue (**Figure 1**), a characteristic pathological feature seen in aplastic anemia (14), rather than cytotoxic myelosuppression caused by the chemotherapy. The pathological features and clinical course of immediate recovery by systemic immunosuppressants exactly mirror several previous case reports of ICI-induced aplastic anemia (10, 11). Aplastic anemia triggered by carboplatin, paclitaxel, or bevacizumab is exceedingly rare (four, eight, and zero cases in the last 10 years, respectively, according to safety reports from the Pharmaceuticals and Medical Devices Agency of Japan (15)). These indicate that severe myelosuppression was caused by the development of ICI-induced aplastic anemia rather than unspecific cytotoxicity by chemotherapy.

Given its rarity, there is currently no established treatment for ICI-induced aplastic anemia. Idiopathic acquired aplastic anemia typically necessitates multiple systemic immunosuppressants, including steroids, anti-thymocyte globulin, and cyclosporine (14). Patients with ICI-induced aplastic anemia can usually not achieve complete response with systemic steroid treatment alone and require multiple immunosuppressants (2, 10, 11). As per the National Comprehensive Cancer Network guidelines, systemic intravenous administration of steroids at a dosage of 1–2 mg/kg once daily should be considered for severe ICI-induced aplastic anemia, followed by other immunosuppressants if the patient does not respond within 7 days of initiation (3). In this case, a lower dose of prednisolone (0.5 mg/kg, 25 mg/body) was administered due to concerns about intestinal perforation, resulting in an immediate improvement in peripheral blood count. In idiopathic acquired aplastic anemia, CH in *DNMT3A* is linked to resistance to systemic steroids (12), indicating potential variations in the efficacy of systemic steroid treatment between patients with idiopathic aplastic anemia and those treated with ICIs.

## 4 Limitation

This case report had several limitations. The etiology of aplastic anemia, including idiopathic and ICI-induced ones, is considered as autoimmune mechanisms given expansion of myeloid cancer candidate gene CH, suggesting clonal selection that allows immune evasion or increased cell survival the marrow environment under immune attack (12, 14, 16). However, we can not conclude by observation of the single case. CH is also known to be accumulated by several other factors including aging (17), DNA damage by cytotoxic drug and radiation (18–20), and bone marrow reconstruction after myeloablative treatment (21). We could not further evaluate dynamic changes of the CH through treatment course in serial buffy-coated white blood cells as well as whole peripheral blood and bone marrow samples. Such additional tissue sampling is beyond our nationwide genomic screening study MONSTAR-SCREEN-2. As establishment of aplastic anemia preclinical models is difficult, larger clinical research of prospective genomic landscapes in ICI-induced plastic anemia is highly warranted. The *DNMT3A* CH was identified only in the blood sample collected when the patient was diagnosed with ICI-induced aplastic anemia. The *DNMT3A* CH clone may have developed when the patient was diagnosed with ICI-induced aplastic anemia. The Clone CH had a variant allele frequency that was too low to be detected when we first evaluated her liquid genomic profiling, and the clone might have expanded under hematopoietic stress when she developed ICI-induced aplastic anemia. Pathological, radiological, and hematological responses were discrepant after systemic steroid (**Figures 1**, **2**; **Supplementary Figure 1**). Lack of pathological responses in bone marrow is sometimes observed even after peripheral blood count recovery (22). The serial MRI in the present case demonstrated the potential improvement of cellularity in the thoracic vertebrae, where we could not evaluate pathological responses by bone marrow biopsy. Given that the present case achieved complete responses of peripheral blood counts sustaining over 600 days even after decreasing doses of systemic steroid (**Figure 2**), we did not re-evaluate pathological characteristics of her bone marrow.

## 5 Conclusion

In conclusion, we longitudinally examined the genomic characteristics of patients who developed aplastic anemia caused by an ICI. Serial genomic profiling using liquid biopsy and HLA typing may assist in diagnosing ICI-induced aplastic anemia.

## Data Availability

The original contributions presented in the study are included in the article/**Supplementary Material**. Further inquiries can be directed to the corresponding author.
