# Supplementary material for: Genomic landscape of immune checkpoint inhibitor-induced aplastic anemia: a case report
Source: Front Oncol. 2026 May 20;16:1801452. doi: 10.3389/fonc.2026.1801452 (PMC13229815; doi:10.3389/fonc.2026.1801452)
Supplement: Supplementary file 1 [file SupplementaryFile1.docx]

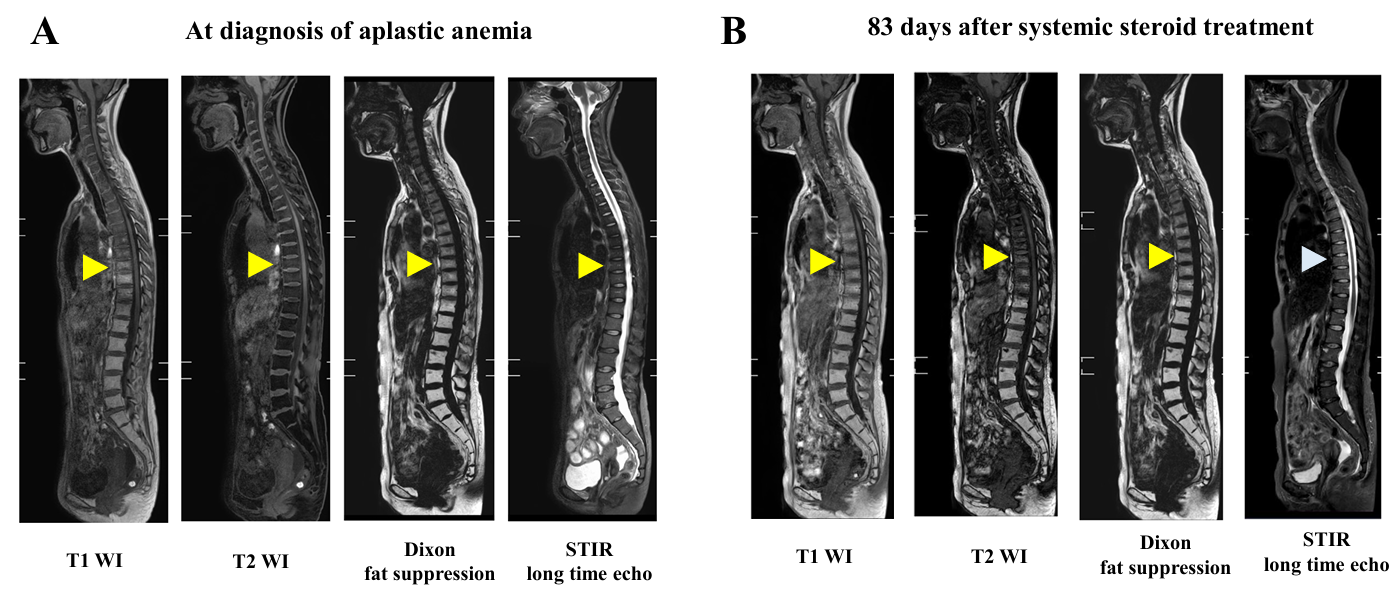


**Supplementary Figure 1. Serial magnetic resonance imaging of vertebrae**

A: Magnetic resonance images at the time of aplastic anemia diagnosis. B: Magnetic resonance images taken 83 days after the diagnosis of aplastic anemia.

The yellow arrowheads indicate the thoracic vertebrae. The light blue arrowhead indicates the improved intensity of STIR long-term echo images 83 days after the diagnosis of aplastic anemia. Abbreviations: STIR, short inversion time inversion recovery.

Supplementary Table 1. Laboratory results for infection, virology, and autoantibody.

| Autoantibody |  |  |  | Virology |  |  |
| --- | --- | --- | --- | --- | --- | --- |
| IgG | 1100 | mg/dl |  | HBs antigen | negative |  |
| IgA | 105 | mg/dl |  | HBs antibody | negative |  |
| IgM | 99 | mg/dl |  | HBc antibody | negative |  |
| Antinuclear antibody | < 40 | titer |  | HCV antibody | negative |  |
| Homogenous | < 40 | titer |  | HIV antibody | negative |  |
| Speckled | < 40 | titer |  | EBV DNA | undetectable |  |
| Nucleolar | < 40 | titer |  | CMV antigen | negative |  |
| Peripheral | < 40 | titer |  | HHV6 DNA | undetectable |  |
| Discrete sp. | < 40 | titer |  | Parvovirus B19 IgG | 0.99 | index |
| Cytoplasmic | < 40 | titer |  | Parvovirus B19 IgM | 0.33 | index |
| Anti-platelet antibody | negative |  |  | Parvovirus B19 DNA | undetectable |  |
| Platelet-associated IgG | 46 | ng/10^7^ cells |  |  |  |  |
| Direct Coombs test | negative |  |  |  |  |  |
| Infection |  |  |  |  |  |  |
| Blood culture | negative |  |  |  |  |  |
| Procalcitonin | 0.12 | ng/ml |  |  |  |  |
